# Assessing the association of rs7574865 STAT4 gene variant and type 1 diabetes mellitus among Egyptian patients

**DOI:** 10.1186/s43141-021-00214-2

**Published:** 2021-08-03

**Authors:** Samar Samir Abdelmajed, Mohamed A. El-Dessouky, Doaa S. SalahElDin, Naglaa Abu-Mandil Hassan, Moushira Erfan Zaki, Somaia Ismail

**Affiliations:** 1 Modern Sciences and Arts University (MSA), Giza, Egypt; 2 Chemistry Department (Biochemistry Division), Faculty of Science, Cairo University, Giza, Egypt; 3 Biological Anthropology Department, Medical Research Division, National Research Centre, Giza, Egypt; 4 Medical Molecular Genetics Department, Human Genetics and Genome Research Division, National Research Centre, Giza, Egypt

**Keywords:** Autoimmune disease, Diabetes type 1, Real-time PCR, rs7574865 polymorphism, Signal transducer and activator of transcription 4

## Abstract

**Background:**

Variants in the signal transducer and activator of transcription 4 (STAT4) gene have an important role in the incident of multiple autoimmune diseases including type 1 diabetes mellitus (T1D). It is a genetically related auto-immune disorder that resulted from T cell-mediated destruction of pancreatic cells that are in control for the production of insulin in the blood. The current study aimed to clarify the role of STAT4 (rs7574865) variant allelic and genotypic variations in the susceptibility to type 1 diabetes among Egyptians by using the real-time PCR.

**Results:**

A total of 100 patients and 100 controls were genotyped for rs7574865, and the biochemical and anthropometric parameters were measured to show that type 1 diabetic patients had significantly higher levels of HbA1c and triglycerides compared to non-diabetic individuals (*P* < 0.05). And genetically, the T allele and GT genotype have a significant correlation with diabetes type 1.

**Conclusion:**

It was confirmed by this study that the rs7574865 T allele and GT genotype have a significant correlation with diabetes type 1 incidence among Egyptian patients.

## Background

Diabetes is a chronic disease that is characterized by an elevated sugar level in the blood that is either due to compromised insulin excretion from the pancreas or cellular resistance to insulin; it is a multifactorial disease, and generally, it is subdivided into three types (type 1 diabetes, type 2 diabetes, and gestational diabetes) [1]. Diabetes mellitus is considered one of the most important diseases globally because of its high morbidity and major side effects. The number of diabetic patients escalates globally up to 451 million among people between 18 and 99 years old. Moreover, it is expected that when we reach 2045, there will be around 693 million people who will have diabetes mellitus [2]. Type 1 diabetes is an autoimmune disorder developed due to autoimmune destruction of pancreatic beta cells [3];, also, it is triggered due to some other factors in genetically susceptible individuals [1, 4]. Type 1 diabetes mellitus (T1D) is not a widely spread kind of diabetes like type 2, as it represents from 5 to 10% of diabetes mellitus patients [5]. Type 1 diabetes also is the most communal metabolic and endocrine disorder in children and adolescents; its incidence is mostly due to genetic disorders. Genome-wide association studies and meta-analyses identified multiple genetic risk factors for T1D [6]. The main genes causing type 1 diabetes mellitus are found mainly within the major histocompatibility complex region (HLA) [7]. Genetics have a crucial role in the susceptibility of diabetes type 1; the most common genes related to type 1 diabetes are CTLA-4, PTPN22, STAT4, STAT3, and IFIH1. Certain genes are commonly found in one group of people than in another, and that is why race and ethnicity affect disease incidence [8]. Signal transducers and activators of transcription (STAT) proteins are activated by many cytokines, the mechanism of STAT4-mediated IL-12 signaling [9–11], and there is a strong evidence that supports a role of IL-12 in autoimmune diabetes [12–14]. The signal transducer and activator of transcription 4 (STAT4) gene is in charge of signal transduction of multiple proinflammatory cytokines [15]; its genetic position is 2q32.2-32.3. STAT4 gene is vital in the development of Th1 cells so it plays an important role in immunity, and that is why it was associated with different immune diseases. This study, therefore, inspects the impact of STAT4 variants on the vulnerability of type 1 diabetes among Egyptians.

## Methods

### Subjects

We recruited 200 un-related Egyptians, 100 T1DM patients who were assured diabetic patients as per the American Diabetes Association (ADA), and 100 healthy individuals. All patients were diagnosed as type 1 diabetic before the age of 15 years and were dependent on exogenously administered insulin. And control was healthy people with no chronic disease such as any other types of diabetes, cardiovascular, renal, and hepatic diseases. No clinical evidence or family history of autoimmune diseases were eligible for inclusion, while non-Egyptians, Egyptian patients with any type of diabetes, and patients with clinical evidence or family history of autoimmune diseases, renal, and hepatic disease were excluded. This research was accepted by the Ethical Committee of the Egyptian Ministry of health and population (No: 3-202027). The subjects were recruited from governmental hospitals and the participants and parents of young participants provided us with a written informed consent, as per the Helsinki Declaration on Human Experimentations guidelines.

### Biochemical and anthropometric measurements

Anthropometric measurements such as body weight, waist circumference (WC), height, and hip circumference (HC) were measured by the regular ways [16], The body mass index (BMI) was calculated by dividing the weight in kilograms by height in meters squared (kg/m^2^), fasting serum triglycerides (TG), high-density lipoprotein cholesterol (HDL-C), low-density lipoprotein cholesterol (LDL-C), very low-density lipoprotein cholesterol (VLDL-C), and glycelated hemoglobin (HbA1c) were measured enzymatically by using commercially available kits.

### Genotyping assessment

Blood samples were obtained from all the subjects in tubes with ethylenediaminetetraacetic acid anticoagulant. Total genomic DNA was extracted from the blood of all subjects using (PREP-RAPID, DNA, extraction kit, by (DNA-technology research and production ) company, Moscow, Russia) according to the manufacturer’s protocol, and to guarantee the quality of the extracted DNA concentrations were measured with Nano Drop (Nano Drop 2000/2000c, Thermo Scientific™). Analysis for the rs7574865 polymorphism was accomplished by using Taqman-probes-based real-time PCR reactions. This analysis was done in a 25-μL reaction with 20 ng of DNA, which was added to 12.5 μL TaqMan Universal Master Mix and 1.25 μL working stock. The samples were re-genotyped to endorse the results of the research. We used the Applied Biosystems Step One Real-time PCR - SDS version 2 software.

### Statistical analysis

Genotypes were calculated by direct counting. We calculated Hardy–Weinberg equilibrium (HWE) by the chi-squared (*χ*^2^) test with expected frequencies derived from the actual frequencies in the SNP of patients and control. Anthropometric measurements and biochemical parameters between genotypes were compared using Student’s t test. The *χ*^2^ to compare between genotype frequencies for patients and control was done by SPSS software for windows (version 16.0; SPSS Inc, Chicago, IL). Moreover, the Hardy-Weinberg equilibrium status of patients and controls were measured by the goodness-of-fit chi-square test. Statistical significance was set at a probability (*P* value < 0.05).

## Results

STAT4 genotype (rs7574865 G>T) frequencies were investigated in all 200 subjects (100 type 1 diabetes patients and 100 controls) as presented in (Table 1). It has been shown that the GT genotype has significantly higher frequency among T1D patients (75% GT and 2%TT, *P* < 0.05), than in control (30.0%) who mostly had wild-type GG genotype, Similarly, the minor allele (T) was most common among patients with percentage (41.3%) in comparison to control (15%) and (*p* = 0.01). Thus the outcomes suggest an association between the GT genotype and type 1 diabetes patients.

**Table 1:** distribution of genotypes and alleles of rs7574865 (G>T) in between type 1diabetic patients and controls

		Group		*P* value
		Control (%)	Patient (%)	
Genotypes	GG	70.0%	23%	0.01
	GT	30.0%	75%	
	TT	0%	2%	
Alleles	G	85%	58.6%	0.01
	T	15%	41.3%	

rs7574865 (G/T) GG: denotes wild type, *TT* homozygous mutant, *GT* heterozygous carriers

As demonstrated in Table 2, the demographics and clinical parameters of the study patients, subjects with the T allele, showed higher mean values for weight, Body mass index, waist circumference, hip circumference, glyclated hemoglobin, triglycerides and low-density lipids, and inferior value of high-density lipids and waist-hip circumference ratio. Furthermore, we assessed the correlation between the STAT4 rs7574865 variant and previously mentioned metabolic and anthropometric parameters, Table 3 showed that there was an association with HbA1C and TG with *p* value < 0.05.Álso the observed genotype frequencies of the SNP was statistically consistent with the expected distributions according to Hardy–Weinberg equilibrium.

**Table 2:** clinical and anthropometric characteristics of study patients

Parameters	GG	GT+TT
	Mean ± SD	Mean ±SD
WT	53.72 ±19.90	55.49 ±20.25
HT	152.22 ±16.60	151.8 ±15.20
BMI	21.85 ±5.44	23.41 ±5.88
WC	71.61 ±17.84	72.2 ±21.50
HC	77.7 ±32.09	81.7 ±28.31
WHC	1.04 ±0.42	0.957 ±0.43
HbA1C	29.14 ±67.94	68.48 ±62.30
TG	123.46 ±62.25	156.82 ±61.59
HDL	50.44 ±19.78	42.29 ±27.8
LDL	71.94 ±36.04	95.70 ±40.12
VLDL	23.96 ±24.36	30.74 ±31.38

Data presented values are expressed as mean ± SD for genotypic classes, GG and GT for rs7574865, *WT* weight, *HT* height, *BMI* body mass index, *WC* waist circumference, *HC* hip circumference, *WHC* waist-to-hip circumference, *HbA1C* glyclated hemoglobin, *TG* triglyceride, *HDL* high-density lipoprotein, *LDL* low-density lipoprotein, *VLDL* very low-density lipoprotein

**Table 3:** *P* values of biochemical and anthropometric parameters regarding rs7574865

Parameters	*p* value
WT	0.933
HT	0.270
BMI	0.353
WC	0.431
HC	0.76
WHC	0.444
HbA1C	0.003*
TG	0.031*
HDL	0.806
LDL	0.778
VLDL	0.635

*P < 0.05 which means there is a significant correlation, T allele carriers vs. non T allele carriers

## Discussion

Type 1 diabetes incidence is due to multiple factors one of the most important factors is genetics, STAT proteins are cytosolic proteins that have a role in the intracellular signaling downstream of the type I and type II cytokine receptors [17–19], former studies and researches showed that type 1 diabetes incidence have been related to different genetic variants among different genes, such as a study on Tunisians showed that a variant of CREM gene, PTPN22, TCRβ, CD3z, ZAP70, and CTLA-4 gene variants correlation with T1D [20–22]. Moreover, it has been confirmed by earlier studies the correlation between the c.49A>G polymorphism of the CTLA4 gene and Egyptian, Lebanese, and Iraqi population and type 1 diabetes incidence [23–25]. Also, another prior study on the STAT4 gene showed confirmed the association of the STAT4 gene and various autoimmune diseases [26, 27] as the STAT4 gene codes a transcription factor that transfers signals induced by type 1 cytokines type1-IFN, IL-12, and IL-23 [28–30]. Moreover, it has a crucial role in the differentiation of T cells into the Th1. An earlier study on mice showed that diabetes type 1 was reduced by the interruption of the STAT4 signaling pathway [31]. And it has been proven earlier that some SNPs at STAT 4 gene contribute to the predisposition of diabetes type 1. This study support that the polymorphism rs7574865 has an association with the incidence of diabetes type 1 among Egyptians. rs7574865 variant, specifically, has been stated to be accompanied by various autoimmune diseases [15], and a preceding study confirmed that there is an association between rheumatoid arthritis and rs7574865 (T) allele among Spanish, Swedish, and Dutch patients and Egyptians [32, 33]. Also, it was proven its association with autoimmune thyroid diseases among the Chinese Han population [34]. Moreover, it was proven the susceptibility with AITD in the Asian population due to STAT4 rs7574865 polymorphism, but not in the African population [35]. Also, it is correlated with the susceptibility of systemic sclerosis [36], and colonic Crohn’s disease [37]. Furthermore, another study confirmed its association among Crete [38], and Mexican populations as well [39]. Also, there was a study that proved rs7574865 variant correlation with lupus among Asian population such as Japanese [40] and Chinese Northern Han population [41], and it was correlated as well with Egyptian population [42]. Most of the earlier studies on Asian and European populations confirmed that rs7574865 has a role in the incidence of diabetes type 1 [43, 44]. Also, a meta-analysis study performed on Caucasians and Asian subjects showed that it was associated with diabetes risk (*P* < 0.5) [45], and there was a study that confirmed the STAT4 was overexpressed from T1D Polish patients [46]. In contrast, a study among the Korean population, the Tunisian and Brazilian population showed no evidence of the association of the variant and T1D [20, 47, 48], while our study showed that the rs7574865 (T) allele is accompanied with increased risk for type 1 diabetes among Egyptians, same as another study on Greek patients [49] and Northeastern Chinese Han population [41, 44]. And we proved that the STAT4 (rs7574865) variant has a significant association and type 1 diabetes, and we found that the (GT) genotype is the most frequent among patients in comparison to the GG genotype as shown previously in (Table 1). We conducted this study because there were almost no studies among Africans and few among the Middle East area specifically the Arab region that shows the correlation between rs7574865 and type 1 diabetes.

## Conclusion

This study showed that there was an association between rs7574865 polymorphism and f type 1 diabetes, these results could help to identify the mechanisms of type 1 diabetes well. And it’s highly recommended that more research should be done in depth to detect the genetics of T1D for treatment goals and providing novel ways for developing beneficial diagnostic and prognostic data for individuals. Moreover, more studies on other autoimmune disorders in Egyptian patients to determine if that variant is related to T1D or immune-related diseases.

## Data Availability

Not applicable

## Abbreviations

T1D: Type 1 diabetes
IL: Interleukin
STAT: Signal transducer and activator of transcription
PCR: Polymerase chain reaction
HbA1c: Hemoglobin A1C
LDL: Low-density lipoprotein cholesterol
HDL: High-density lipoprotein cholesterol
VLDL: Very low-density lipoprotein cholesterol
WC: Waist circumference
HC: Hip circumference
BMI: Body mass index
CTLA-4: Cytotoxic T-lymphocyte-associated protein 4
PTPN22: Protein tyrosine phosphatase non-receptor type 22
IFIH1: Interferon induced with helicase C domain 1
HLA: Human leukocyte antigen
TNF: Tumor necrosis factor
HWE: Hardy–Weinberg equilibrium
